# Pulmonary and intestinal tuberculosis with COVID-19 complicated with fluidopneumothorax and colovesical fistula: The importance of diagnosis complexity in line with clinical setting

**DOI:** 10.1016/j.radcr.2024.04.042

**Published:** 2024-05-14

**Authors:** Soedarsono Soedarsono, Sri Sarwosih Indah Marthaty, Caesar Rozaq Auditiawan, Anita Widyoningroem

**Affiliations:** a Sub-pulmonology Department of Internal Medicine, Faculty of Medicine, Hang Tuah University, Surabaya, Indonesia; b Dr. Soetomo Academic General Hospital, Surabaya, Indonesia; c Dr. Ramelan Navy Hospital, Surabaya, Indonesia; d Department of Pulmonology and Respiratory Medicine, Faculty of Medicine, Airlangga University, Surabaya, Indonesia; e Department of Radiology, Faculty of Medicine, Airlangga University, Surabaya, Indonesia

**Keywords:** Colovesical fistula, COVID-19, Hydropneumothorax, Intestinal tuberculosis, Pulmonary tuberculosis

## Abstract

The complication of hydropneumothorax and colovesical fistula is rare, especially in patients with tuberculosis (TB) and COVID-19. This particular situation poses a management difficulty, and can significantly threaten the patient's life without a clear diagnosis and timely treatment. We report a 28-year-old woman with pulmonary and intestinal TB with COVID-19 complicated with hydropneumothorax and colovesical fistula (CVF) which worsened her condition. Treatment for this patient was given according to the diagnosis. Her condition improved and she was discharged after 30 days of hospitalization, while elective surgery for CVF was not performed because there were no clinical symptoms complained of by this patient after completing TB treatment (9 months after hospital discharge). This case report highlights the importance of considering pulmonary and intestinal TB with COVID-19 as the cause of hydropneumothorax and CVF. Early and complex diagnosis is essential for proper management, as well as the efficacy of medical therapy and treatment for controlling such advanced stages of the disease. A complex condition with many symptoms can overlap with other diseases. Clinicians should consider the clinical symptoms, radiological imaging, and standard or supporting examination for accurate diagnosis to find the etiology of the diseases. Complete treatment for TB should be considered as the treatment choice (nonsurgical therapy) for CVF caused by TB before deciding on surgical intervention.

## Introduction

Tuberculosis (TB) caused by *Mycobacterium tuberculosis* and Coronavirus Disease 2019 (COVID-19) caused by severe acute respiratory syndrome coronavirus 2 (SARS-CoV-2) are infectious diseases that have the same manifestations including cough, fever, and shortness of breath. COVID-19 has acute symptoms, while TB is chronic [1,2]. Indonesia is ranked second country with the highest TB burden, accounting for 10% of the world's TB cases in 2022 [3]. TB problems in Indonesia include pulmonary TB (PTB) and extra-pulmonary TB (EPTB). EPTB accounted for approximately 8-24% of the total TB cases, globally [4]. An estimated 11% of patients with EPTB were diagnosed with abdominal TB and 1%-2% were intestinal TB [5]. Intestinal TB manifests in either ulcerative, hypertrophic ulcerative, or fibrous structuring types [6].

Tuberculosis treatment in the later stage is challenging and its treatment becomes more difficult and expensive. High severity and mortality of TB occur with delayed diagnosis, inadequate therapy, coinfection (eg. COVID-19), and complications [7]. Hydropneumothorax is a critical condition when there is an abnormal presence of air and fluid in the pleural space [8,9]. Hydropneumothorax is a complication of pulmonary TB and causes significant morbidity [8,10]. Intestinal TB can lead to serious complications such as intestinal obstruction, perforation, intestinal fistula, intra-abdominal effusion, and gastrointestinal bleeding [11]. A fistula is an abnormal anatomic connection between 2 epithelialized surfaces [12]. A colovesical fistula (CVF) is an abnormal connection between the colon and urinary bladder. Although they are uncommon, CVFs can cause significant morbidity, affect quality of life, and may lead to death, usually secondary to urosepsis. Although a CVF can be diagnosed clinically, imaging and endoscopy are often required to delineate the extent of a fistula and to elucidate its etiology [13]. TB is a less common cause of CVF [14]. Spontaneous development of CVF is extremely rare in the era of highly effective anti-TB treatment [15].

The complication of hydropneumothorax and especially CVF is rare in patients with TB and COVID-19. This particular situation poses a management difficulty, and can significantly threaten the patient's life without a clear diagnosis and timely treatment [15,16]. We report a woman with pulmonary and intestinal TB with COVID-19 complicated with hydropneumothorax and CVF which worsened her condition.

## Case presentation

A 28-year-old woman was referred and admitted to our hospital from March 23 to April 26, 2022 (in the COVID-19 pandemic era). This patient presented dyspnea since 1 day, recurring cough with white phlegm for 2 months, fever for the last 4 months, loss of appetite, weight loss by 10 kg for the last 2 months (34 kg to 24 kg), and urinating brownish yellow (faecaluria) since 4 months. This patient has no diabetes mellitus, hypertension, cardiac disease, asthma, history of TB treatment, and allergy. The patient was not a smoker, she also reported no close contact with COVID-19, close contact with TB was unknown, and no history of previous travel. This patient has been twice vaccinated for COVID-19. The result of the HIV test from the previous hospital showed non-reactive.

The general condition appeared to be moderate, consciousness compos mentis, blood pressure 102/64 mmHg, pulse 122 times per minute regularly, respiratory rate 28 times per minute, oxygen saturation 96% with a simple mask (SM) oxygen device of 6 lpm, with a temperature of 37.2 C. Physical examination of the head and neck revealed dyspnea, no cyanosis or icicles, no deviation of the trachea, no enlarged lymph nodes, or increased jugular venous pressure.

Physical examination of the chest revealed asymmetrical chest movement, lagging left side. Tactile fremitus in the lower 2/3 of the left lung decreases. Hypersonor percussion on the lower 2/3 of the left hemithorax, sonor on the upper 1/3 of the left hemithorax, and sonor on the right hemithorax. Auscultation showed that breath sounds disappeared in the lower 2/3 of the left side and vesicular in the right hemithorax and upper 1/3 of the left lung, without crackles (rhonchi) or wheezing.

Physical examination of the abdomen revealed no collateral veins or cicatrix were visible. Auscultation bowel sounds were within normal limits, tympanic percussion, no palpation of tenderness, while liver and spleen were not palpable. There was pain when urinating, there was also vaginal discharge (fluor albus) with a lot of mucus and smell. Examination of the extremities revealed warm dry red acral, no edema, and CRT less than 2 seconds, superior motor 5/5, inferior motor 5/5.

Laboratory examination showed an increase in infection markers seen in leukocytosis (11,050 per µL) and chemical C-reactive protein (CRP) at 11.35 mg/dL. Thrombocytosis (777,000 per µL) was also seen in the patient. There is also hypoalbuminemia 3.03 g/dL. The results of blood gas analysis with O_2_ room air showed pH 7.47, pCO2 36 mmHg, pO_2_ 92 mmHg, HCO3 26.2 mmol/L, BE 2.5, and SpO_2_ 98% with the impression of respiratory alkalosis.

**Fig. 1:**
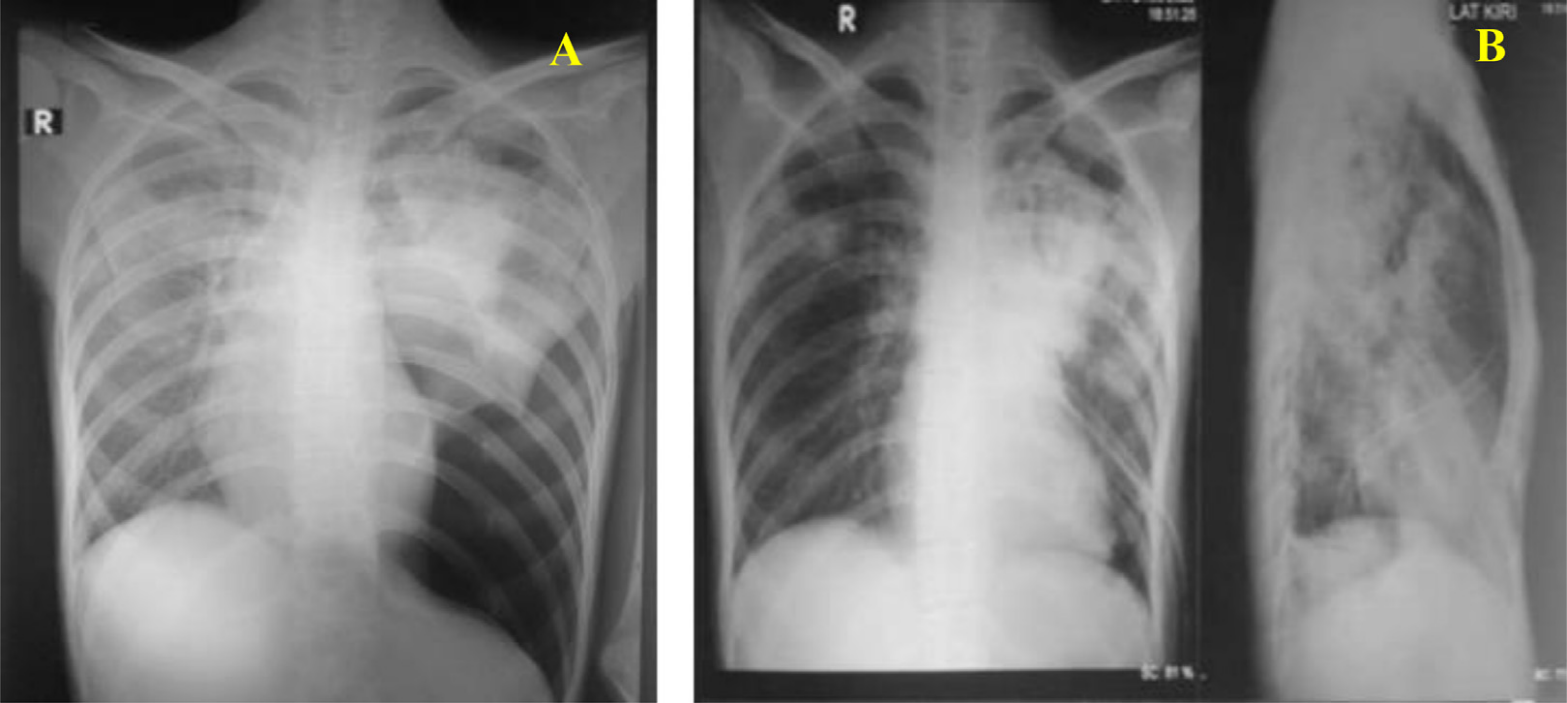
The result of AP chest X-ray of the patient on the first day of hospitalization. (A) an impression of pneumothorax was obtained and (B) a chest tube was placed.

**Fig. 2:**
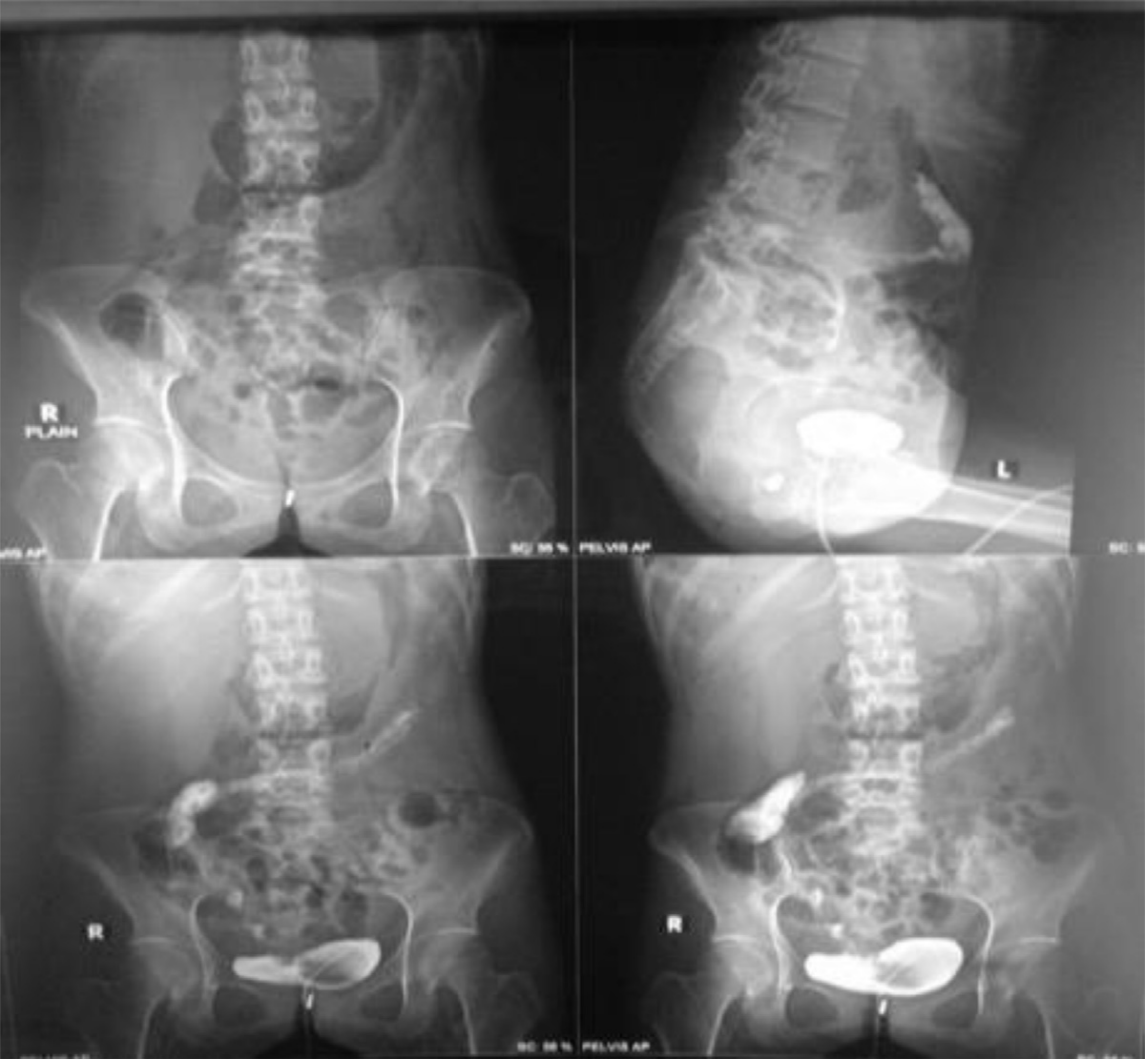
The result of the cystography showed a colovesical fistula.

Chest X-ray (CXR) on the first day of hospitalization (Fig. 1) showed the impression of a pneumothorax in the lower 2/3 of the left lung and compression of the mediastinum in the contralateral direction (A), then a chest tube was placed (B). The cystography results on 3 months before hospitalization showed the impression of a CVF (Fig. 2).

**Fig. 3:**
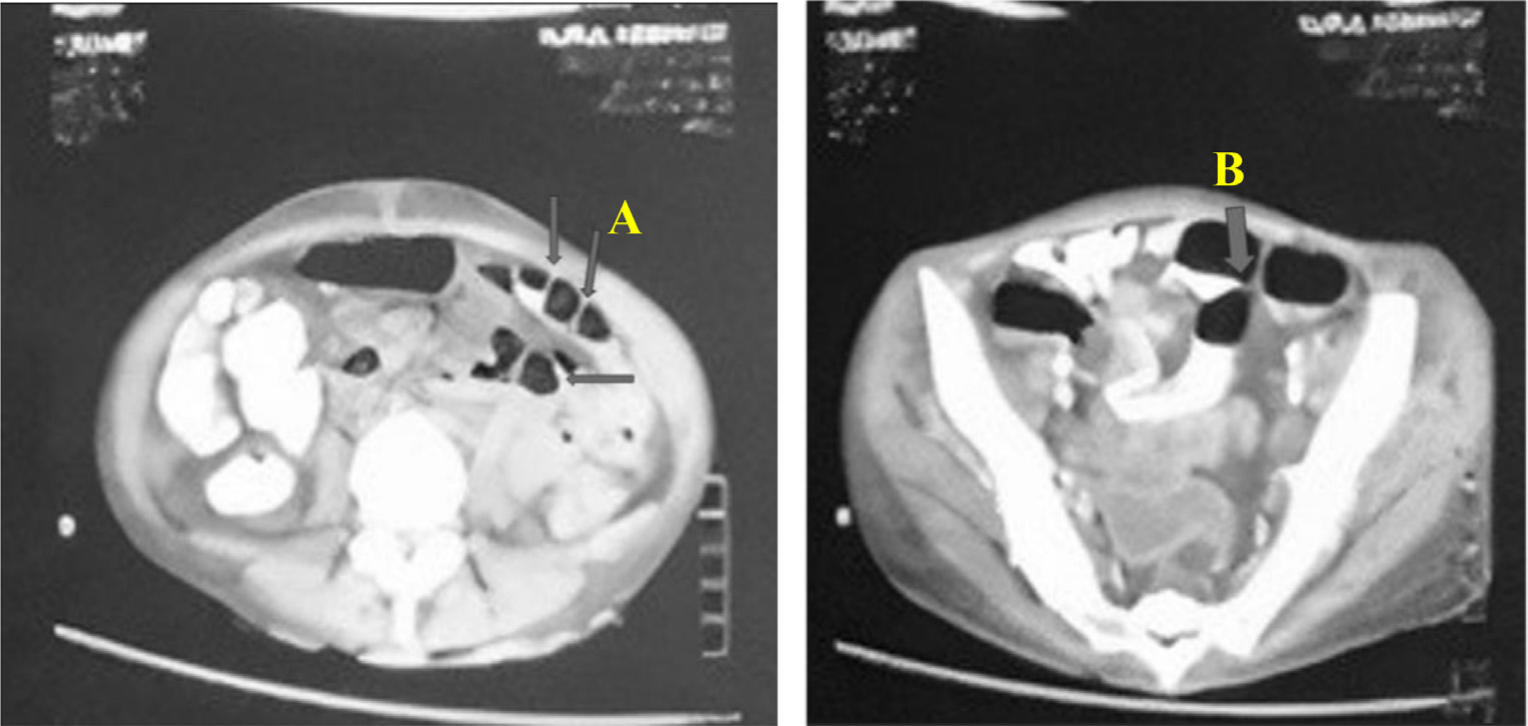
The result of abdominal CT scan on seventh day of hospitalization. (A) diffuse wall thickening and (B) fistula.

Diagnosis of hydropneumothorax and CVF were confirmed according to the chest x-ray and cystography. Dyspnea was improved while fever still fluctuated on the 2nd day of hospitalization. Sputum and stool were then examined for molecular rapid test using GeneXpert MTB/RIF on the 3rd day of hospitalization. This patient also underwent an abdominal computer tomography scan (CT scan) which showed intestinal TB (Fig. 3). CT scan result showed the presence of a fistule between the right posterolateral wall of the bladder and the ileo-caecal wall through which the catheter passes. The catheter tip is distal and within the cecum's lumen. Diffuse thickening of the walls of the ileum, ileocaecal, descending colon, and rectosigmoid accompanied by fat stranding and adhesions of the surrounding bowel walls, thickening of the peritoneum, and multiple mesenterial lymphadenopathy can be differential diagnosis with (1) Intestinal TB, (2) Inflammatory bowel diseases. CT scan also found diverticulosis of the small bowel wall and hepatomegaly.

On the fifth day of hospitalization, dyspnea was improved significantly, and oxygen saturation was 97% with a nasal cannula 2 lpm simple. The patient was planned to have a thorax HRCT scan on the 12th day of hospitalization, but she reported a persistent cough, cold, and fever. Therefore, a reverse transcriptase-polymerase chain reaction (RT-PCR) was carried out. On the 13th day of hospitalization, the examination of RT-PCR showed positive for SARS-CoV-2. She was then admitted to the isolation room for COVID-19, with oxygen saturation of 96% with O_2_ room air. Treatment for COVID-19 was given according to the standard guidelines for COVID-19 treatment in our hospital. Antiviral was not prescribed for this patient due to abnormal laboratory results (leukocytosis, thrombocytosis, hypoalbuminemia, increased D-dimer and CRP levels) and no oxygen delivery device (oxygen saturation 96% with O_2_ room air). Albumin, folic acid, N-acetyl cysteine, vitamin C, vitamin D, and zinc were also prescribed for this patient. Anticoagulant 1 × 0.6 cc subcutaneously was also prescribed according to the presence of hypercoagulopathy (D-dimer 3970 ng/mL).

GeneXpert MTB/RIF results both for sputum and stool samples also showed *Mycobacterium tuberculosis* detected and rifampicin resistance not detected. Pulmonary TB and intestinal TB diagnosis were confirmed according to the results of GeneXpert MTB/RIF and abdominal CT scan. The current confirmed diagnosis was pulmonary TB, hydropneumothorax, intestinal TB, CVF, and COVID-19. Anti-TB treatment consisted of rifampicin 225 mg, isoniazid 125 mg, pyrazinamide 750 mg, and ethambutol 400 mg was prescribed for this patient to treat pulmonary and intestinal TB. A chest tube on Water Seal Drainage (WSD) suction was placed for hydropneumothorax management, while elective surgery was being scheduled. Laboratory examination was also carried out to evaluate the disease progress (Table 1).

**Table 1:** Summary of laboratory examination.

Day of hospitalization	Reference Range	1	2	7	12	13	16	20
GeneXpert MTB/RIF sputum sample	Neg					MTB detected/ RIF res not detected		
GeneXpert MTB/RIF stool sample	Neg					MTB detected/ RIF res not detected		
RT-PCR	Neg					Pos		
HIV test	NR	NR						
Leucocyte (10^3^/µL)	4-10	11.05		15.76	17.81		10.03	
Thrombocyte (10^3^/µL)	150-450	777		560	531			
Albumin (g/dL)	3.4-5.0	3.03		2.56	2.71		3.38	
D-dimer (ng/mL)	<500						3,970	2,320
CRP (mg/dL)	0-1	11.35						9.1

MTB, *Mycobacterium tuberculosis;* Neg, negative; NR, non reactive; Pos, positive; RIF res, rifampicin resistant.

**Fig. 4:**
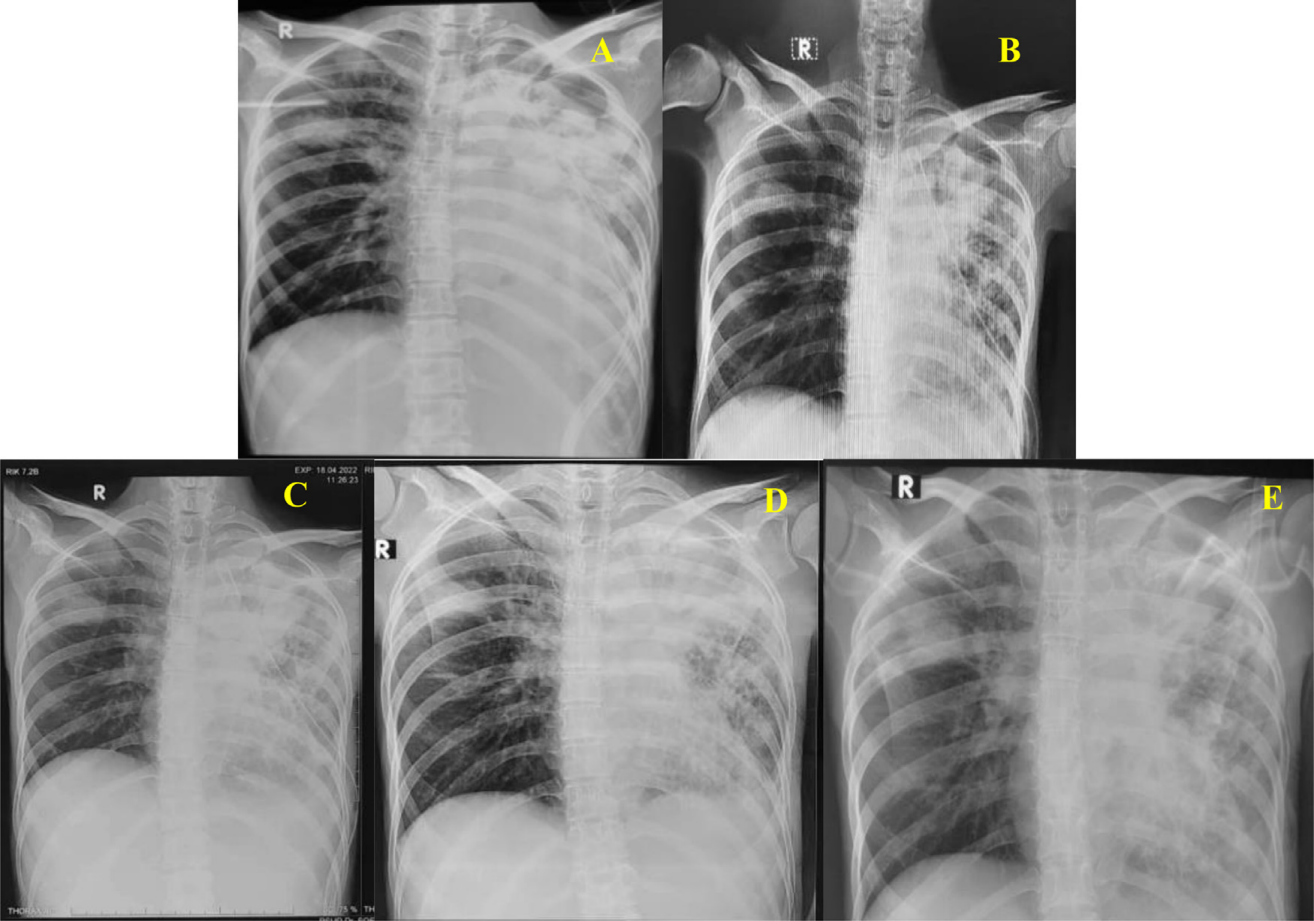
Serial chest x-ray on hospitalization (A. Day 16; B. Day 24; C. Day 26; D. Day 27; E. Day 30).

Chest X-ray on the 16th day of hospitalization showed that the lungs were more inflated. The lungs expanded completely on the 24th day of hospitalization, and then clamping the drain for 2 × 24 hours was carried out. The results of the post-clamp photo show that the lungs were still fully expanded. After 2 × 24 clamps were applied and the lungs remained inflated, a chest tube aff was performed on the 27th day of hospitalization. Re-evaluation CXR was taken on the 30th day of hospitalization after removing the chest tube. This serial CXR is presented in Fig. 4.

The condition of this patient has improved subjectively and objectively, she was discharged and started self-isolation for COVID-19 after 30 days of hospitalization. Surgery for CVF was being scheduled and will be carried out after RT-PCR was negative for SARS-CoV-2. This patient has taken the anti-TB drugs regularly and completed the TB treatment although a sputum smear examination was not performed due to her move to another province. She said that her condition was significantly improved and had no clinical symptoms which she complained about after hospital discharge for 9 months. She also said that the elective surgery was not performed.

## Discussion

The complication of hydropneumothorax and especially CVF is rare in patients with TB and COVID-19. This particular situation poses a management difficulty, and can significantly threaten the patient's life without a clear diagnosis and timely treatment [15,16]. This 28-year-old patient has no diabetes mellitus, hypertension, cardiac disease, asthma, history of TB treatment, or allergy. The patient was not a smoker, she also reported no close contact with COVID-19, close contact with TB was unknown, and no history of previous travel. This patient has been twice vaccinated for COVID-19. The result of the HIV test from the previous hospital showed non-reactive. At first time this patient was admitted to our hospital, she presented dyspnea, cough with white phlegm, fever, loss of appetite, weight loss, and faecaluria. The patient's condition was very complex with many symptoms overlapping with other diseases.

Risk factors for TB disease are HIV positive and other immunocompromised diseases, long-term use of immunosuppressant drugs, smokers, alcohol consumption, children <5 years and the elderly, close contact with active TB cases, in settings with a high risk of infection with TB (e.g. community and long-term care facilities), and health care workers. Symptoms of TB disease depend on the location of the lesion, and the clinical manifestations including cough for >2 weeks, cough with phlegm, hemoptysis, chest pain, dyspnea, malaise, weight loss, loss of appetite, fever, and night sweats [17].

This patient was diagnosed with a new case of pulmonary TB according to the GeneXpert MTB/RIF result from a sputum sample (*Mycobacterium tuberculosis* detected and rifampicin resistance not detected), TB symptoms, no close contact with TB cases, and no history of previous TB treatment. This patient was diagnosed with intestinal TB according to the GeneXpert MTB/RIF result from a stool sample (*Mycobacterium tuberculosis* detected and rifampicin resistance not detected), and a CT scan of the abdomen showed diffuse thickening of the walls of the ileum, ileocaecal, descending colon and rectosigmoid accompanied by fat stranding and adhesions of the surrounding bowel walls, thickening of the peritoneum, and multiple mesenteric lymphadenitis which can lead to intestinal TB [6,17,18]. A previous study reported that the examination of CT abdomen and pelvis with oral and intravenous contrast in a patient with intestinal TB revealed thickening of the terminal ileum and cecum, to a lesser extent to the ascending and transverse colon [19]. Another study reported that the radiologic examination in patients with intestinal TB showed bowel wall thickening, fibrofatty proliferation, and abdominal lymphadenopathy [20]. CT scan images can be used to differentiate Crohn's disease from intestinal TB [19]. The barium enema and CT scan images of intestinal TB can be seen in Fig. 3.

*Mycobacterium tuberculosis* complex organisms cause TB primarily in the lungs (pulmonary TB) but can also affect other organs, causing extrapulmonary TB (EPTB) such as the pleura, lymph nodes, bones, and other extra-pulmonary organs. EPTB can be either primary (at the site of initial infection) or secondary (disseminated), which usually occurs due to hematogenous or lymphatic spread of bacteria from the primary organ, reactivation of latent TB, ingestion of infected sputum, or spread locally from adjacent organs [17,21,22]. The clinical manifestations of intestinal TB were non-specific and sometimes mimicked other conditions including inflammatory bowel disease, colonic malignancy, or other gastrointestinal infections, making it difficult to be diagnosed and differentiated from other diseases as well as prone to misdiagnosis [6,11,23]. Weight loss is one of the most common symptoms that occurs in patients with intestinal TB due to various causes such as chronic inflammatory processes, decreased intake, and impaired absorption. Weight loss can be accompanied by mild to moderate anemia. Most intestinal TB patients also experience irregular low-grade fever, with body temperature between 37.5 and 38.5°C, accompanied by night sweats. Other gastrointestinal symptoms also often occur such as chronic diarrhea, constipation, and decreased appetite. On physical examination, it is often found ascites and palpable abdominal mass, especially in the right lower quadrant area, and splenomegaly [24]. Manifestations may be nonspecific and show similarities to other gastrointestinal disorders, such as Crohn's disease, peptic ulcer, malignancy, sarcoidosis, fungal infections, or idiopathic granulomatous gastritis [25,26].

Anti-TB treatment consisted of rifampicin 225 mg, isoniazid 100 mg, pyrazinamide 500 mg, and ethambutol 350 mg were prescribed for this patient to treat pulmonary and intestinal TB. This was according to the standard regimen for new TB cases recommended by the WHO is 2RHZE/4RH (2 months of intensive phase using rifampicin, isoniazid, pyrazinamide, and ethambutol and 4 months of continuation phase using rifampicin and isoniazid) [6,17,18]. The regimen for intestinal TB treatment was the same recommended regimen as for pulmonary TB [6,17,18]. Anti-TB drugs should be prescribed in full doses to complete the treatment, while surgery is the second option to treat intestinal TB with complications [27,28].

This patient has been confirmed for COVID-19 pneumonia with moderate severity. The examination of RT-PCR was carried out on the 12th day of hospitalization due to the persistent cough, cold, and fever, and showed positive for SARS-CoV-2. Symptoms of COVID-19 appeared after an incubation period of around 5 days. The most frequent symptoms are fever, cough, weakness, or myalgia [29]. Examination of CRP on the first day of hospitalization showed elevated CRP levels (11.35 mg/dL). CRP is an acute inflammatory protein that increases up to 1,000-fold at sites of infection or inflammation [30]. Elevated CRP levels were commonly reported in TB patients and COVID-19 patients. CRP levels increase in the event of disease progression and will decrease with successful treatment indicating an effective therapeutic response [31,32].

In this patient, left hydropneumothorax was confirmed from clinical data and supporting examinations. This was the complication of pulmonary TB. Hydropneumothorax is a critical condition when there is an abnormal presence of air and fluid in the pleural space [8,9]. This patient had symptoms of dyspnea, asymmetrical chest movement, lagging left, palpable fremitus decreased 2/3 of the lower parenchyma, hyper resonance on percussion of the lower 2/3 of the left hemithorax, and auscultation of breath sounds were lost in the lower 2/3 of the left hemithorax which leads to the presence of pneumothorax. CXR result was suggestive of left hydropneumothorax, and pleural fluid analysis showed protein 4.46 g/dL (±0.9 g/dL), glucose 32.6 mg/dL (SD ± 41.56), and a dominant polymorph indicating the direction of exudative hydropneumothorax caused by TB. Left hydropneumothorax was treated with the placement of a chest tube and Water Seal Drainage (WSD) suction.

Most patients with hydropneumothorax caused by TB presented with symptoms and signs of cardiorespiratory distress along with cough, anorexia, and weight loss. TB is the most common etiology in 80.7% of patients with hydropneumothorax [8,24]. In countries with a high prevalence of TB, a ruptured cavity of TB can be a cause of pneumothorax or hydropneumothorax. Hydropneumothorax can be diagnosed according to pleural fluid analysis, pleural fluid culture, CXR, or thorax CT scan [33]. Thorax CT scan is important for etiological diagnosis because it is easier and faster than pleural fluid analysis, therefore it can be used as an initial examination. A chest tube (or intercostal drain) is the most definitive initial treatment of a hydropneumothorax. WSD is used in various hospitals to treat cases of pleural effusion, pneumothorax, or hydropneumothorax [9]. An intercostal drain (ICD) device is inserted for drainage. In cases with TB etiology, ICD insertion may take longer, up to 30 days [33].

Pneumothorax is a serious and relatively frequent complication of human immunodeficiency virus (HIV) infection that may be associated with increased morbidity and mortality and may prove difficult to manage, especially in patients with acquired immunodeficiency syndrome (AIDS). The incidence of spontaneous pneumothorax in HIV-seropositive individuals is estimated to be about 2%-5% of overall total cases. Spontaneous pneumothorax occurs 450 times more frequently in patients with AIDS versus the general population [34]. This patient was negative for HIV according to the result of the HIV test from a previous hospital which showed non-reactive.

Colovesical fistula was also obtained from clinical and supporting examinations in this patient. The patient had diarrhea and fecaluria for 4 months. CT scan and cystography showed a fistula between the right posterolateral wall of the bladder and the ileocaecal wall. This fistula can be caused by intestinal TB infection [35]. CVF is a complex colorectal condition, which carries significant morbidity and negatively impacts quality of life. Proper evaluation starting with an accurate history and physical examination is the initial step in the management of the patients. Prompt recognition of CVF by imaging study is the initial necessary phase before treatment [12]. The common clinical manifestation of CVF is recurrent urinary tract infections. Pneumaturia (50%-70%) and faecaluria (up to 50%) are common presenting symptoms and are considered pathognomic of a CVF [36]. Diagnostic investigations aim at confirming the presence of a fistula [37]. Examinations for CVF included a CT scan, colonoscopy, cystoscopy, barium enema, poppy seed test, and MRI [37,38]. CT scans are diagnostic in 90%–100% of patients with a CVF [36]. CVF can be caused by intestinal TB infection [35]. TB is a less common cause of CVF [14]. Spontaneous development of CVF is extremely rare in the era of highly effective anti-TB treatment [15].

The condition of this patient has improved subjectively and objectively, she was discharged and started self-isolation for COVID-19 after 30 days of hospitalization. This patient has taken the anti-TB drugs regularly and completed the TB treatment although a sputum smear examination was not performed due to her move to another province. This patient said that her condition was significantly improved and no clinical symptoms which she complained about after hospital discharge for 9 months, and elective surgery was not performed. This improvement suggested the healing of CVF without surgical intervention which occurred due to the completion of TB treatment.

Staged surgical procedures remain the mainstay of CVF treatment [37]. However, CVF can sometimes be managed conservatively. A previous study reported the complete healing of CVF without surgical intervention, conservative management of CVF should be considered before surgical decision. In patients where clinical evidence of infection was found, the patient can be treated with systemic antibiotics. Treatment of CVF includes non-surgical and surgical strategies. The non-surgical treatment is reserved for selected patients who are unfit for surgery. In consideration of the etiology and the comorbidity, surgical management can be a demolitive or a conservative treatment, depending on whether a bowel resection is performed or not [36].

## Conclusion

This case report highlights the importance of considering pulmonary and intestinal TB with COVID-19 as the cause of hydropneumothorax and CVF. Early and accurate diagnosis is essential for proper management, as well as the efficacy of medical therapy and treatment for controlling such advanced stages of the disease. A complex condition with many symptoms can overlap with other diseases. Clinicians should consider the clinical symptoms, radiological imaging, and standard or supporting examination for accurate diagnosis to find the etiology of the diseases. Complete treatment for TB should be considered as the treatment choice (nonsurgical therapy) for CVF caused by TB before deciding on surgical intervention.

## Limitation

The limitation of this study is the evaluation of TB treatment completion was only according to the clinical evaluation after the patient completed TB treatment using anti-TB drugs. The decision to not perform surgery for CVF was also because there were no clinical symptoms complained of by this patient after completing TB treatment (9 months after hospital discharge). Sputum smear examination for evaluation of TB treatment and the radiological imaging to evaluate CVF were not performed because this patient moved to another province.

## Ethics approval and consent to participate

We are exempt from ethical approval as it is not required in our hospital for a single case report, but informed consent has been obtained from the patient.

## Patient consent

The authors certify that they have obtained all appropriate patient consent forms. In the form, the patient has given her consent for her images and other clinical information to be reported in the journal. The patient understand that name and initials will not be published and due efforts will be made to conceal identity, but anonymity cannot be guaranteed.
